# EBOVAC-Salone: Lessons learned from implementing an Ebola vaccine trial in an Ebola-affected country

**DOI:** 10.1177/1740774518780678

**Published:** 2018-06-12

**Authors:** Thomas Mooney, Elizabeth Smout, Bailah Leigh, Brian Greenwood, Luisa Enria, David Ishola, Daniela Manno, Mohamed Samai, Macaya Douoguih, Deborah Watson-Jones

**Affiliations:** 1 London School of Hygiene & Tropical Medicine, London, UK; 2 College of Medicine and Allied Health Sciences, University of Sierra Leone, Freetown, Sierra Leone; 3 University of Bath, Bath, UK; 4 Janssen Pharmaceutical Companies of Johnson & Johnson, Leiden, The Netherlands; 5 Mwanza Intervention Trials Unit, National Institute for Medical Research, Mwanza, Tanzania

**Keywords:** Ebola, vaccine, trial, Sierra Leone, lessons, epidemic, recommendations

## Abstract

**Background/aims:**

During the 2014–2016 West African Ebola epidemic, clinical trials were fast-tracked in order to identify prophylactic vaccines and experimental treatments that might be useful in preventing or treating Ebola. These trials included the ongoing EBOVAC-Salone study, which was established and implemented in Sierra Leone to assess the safety and immunogenicity of the Ad26.ZEBOV/MVA-BN-Filo prime-boost Ebola vaccine regimen.

**Methods:**

This article describes the experiences of the EBOVAC-Salone research team in setting up and implementing the trial, and provides recommendations for research teams aiming to conduct clinical trials in future outbreak situations.

**Results:**

Establishing a clinical trial during an outbreak brought some unique challenges, including those related to trial design and the regulatory environment, operational issues, and community engagement. The situation was further complicated by the weak infrastructure and limited experience of clinical trials in Sierra Leone. However, operating in an outbreak context also brought some benefits to the research team, including strong stakeholder support. The EBOVAC-Salone study recruited participants both during and after the outbreak, leading to additional challenges to trial implementation during the post-outbreak transition.

**Conclusion:**

Many lessons have been learned about setting up and implementing a clinical trial during a devastating Ebola epidemic, and some of the experiences of the EBOVAC-Salone team were mirrored by those of other researchers operating in the region. Common to several of these research groups is a recommendation that research should be more closely incorporated into outbreak response planning, which could expedite the establishment of timely and appropriate research projects. We recommend that the lessons learned by researchers during the West African Ebola epidemic are built into programmes and strategies to improve the responses to future epidemics, wherever they occur.

## Background

The scale of the 2014–2016 West African Ebola outbreak was unprecedented. Over 28,000 people were infected across Guinea, Liberia, and Sierra Leone, 11,300 of whom died.^1^ As part of the global response, clinical trials of experimental treatments and prophylactic vaccines were fast-tracked for testing.

In December 2014, the Innovative Medicines Initiative awarded funding from the Ebola + programme to the EBOVAC1 consortium of research institutions in partnership with the Janssen Pharmaceutical Companies of Johnson & Johnson (Janssen) to support the development and evaluation of a prime-boost prophylactic Ebola vaccine regimen.^2,3^ The EBOVAC-Salone study in Sierra Leone, which is still ongoing, is part of the EBOVAC1 project.

The EBOVAC-Salone study is assessing the safety and immunogenicity of the Ad26.ZEBOV/MVA-BN-Filo prime-boost regimen in a population affected by Ebola and is being carried out in Kambia District in northern Sierra Leone. It is coordinated by the London School of Hygiene & Tropical Medicine, in collaboration with the College of Medicine and Allied Health Sciences in Sierra Leone, and is sponsored by Janssen. The Innovative Medicines Initiative is also funding the related Ebola Vaccine Deployment, Acceptance and Compliance consortium which supports communications, community engagement and enabling technologies for the EBOVAC-Salone trial.^4^

Collaborations for the EBOVAC-Salone study were established quickly between November 2014 and March 2015 in an emergency context in which the Ebola epidemic was still claiming lives across Sierra Leone. Vaccination of adult trial participants began on 9 October 2015. The study is ongoing and has recruited and vaccinated participants both before and after the end of the Ebola epidemic, declared by the World Health Organization on 7 November 2015.

This article offers observations on some of the challenges encountered and lessons learned while setting up and recruiting participants into a clinical trial during and after an epidemic, and makes recommendations on how these could be addressed in the future should a similar public health emergency arise. Several research groups have published their own experiences of conducting clinical trials during the Ebola outbreak, and the literature paints a picture of the context within which the EBOVAC-Salone trial was established. A number of researchers have described the difficulty of designing a robust yet ethical trial during an outbreak^5,6^ and then having to amend or abandon their original study objectives as the epidemic waned.^5,7^ Others highlighted the limitations imposed by the infrastructure of the country in which they were operating.^5^ The challenges were not just for researchers: the burden on local ethics committees, dealing with huge increases in workload, was also noted.^7–9^

While many of the challenges and lessons described in this article have been shared by other researchers, this article makes a novel contribution to the literature as one of the few trials to enrol participants both during and after the epidemic. This gives the authors an additional perspective into issues such as community engagement, participant recruitment and trial design, all of which were affected by the end of the outbreak.

## Methods and results

### Trial design

The design of the EBOVAC-Salone study was revised on several occasions in response to the evolution of the Ebola epidemic. During the epidemic’s peak in 2014, an 80,000-person, individually randomised, controlled trial was considered briefly that aimed to determine the efficacy of the Ad26.ZEBOV/MVA-BN-Filo regimen compared to a control vaccine. However, administration of a placebo during a viral outbreak with such a high case-fatality rate had never been done before, and the ethics of this were hotly debated.^10^

Alternative trial designs were considered, including a cluster-randomised trial and a stepped-wedge design.^11^ In January 2015, taking into account ethical concerns, the epidemic circumstances, field conditions, and statistical considerations, the trial was redesigned as a large cluster-randomised trial of approximately 800,000 participants to be randomised to receiving either immediate or delayed vaccination. Regulatory authorities in Sierra Leone requested a staged approach to recruitment to first determine the safety of the vaccine, with initial enrolment of 40 adults who were to be offered immediate vaccination (Stage 1), followed by enrolment of 400 adults (Stage 2) before commencing the main cluster-randomised trial.^11^

Site visits and mathematical modelling were used to help determine both the sample size and geographical location of the study. By the first quarter of 2015, the epidemic was slowing and it was important to determine whether there would be a sufficient number of cases to determine vaccine efficacy. Kambia District in northern Sierra Leone was chosen as the site for the study because no other prophylactic vaccine trial was underway in that part of the country and because modelling data predicted a 50% probability of the epidemic being over in Kambia District by October 2015, compared to a 75% likelihood in Port Loko and Western Area, the other districts under consideration, by August 2015.

However, the epidemic declined at a faster rate than expected during the first quarter of 2015 throughout Sierra Leone. By May 2015, it was clear that the study would be unable to determine vaccine efficacy. The cluster-randomised trial protocol was therefore abandoned before it was approved by the regulatory authorities, and the primary objective for the study was changed to determination of the safety and immunogenicity of the regimen,^11^ with an estimated sample size of approximately 3500 participants. Stage 1 was kept as open label vaccination of 40 healthy adult participants. In December 2015, following the end of the outbreak, the use of placebo was reintroduced to the study design. Stage 2 was formally amended to a double-blind randomised controlled trial assessing the safety and immunogenicity of the vaccine regimen, with follow-up for 12 months and a sample size of approximately 730 participants. Enrolment of 400 adults (randomised in a 3:1 ratio of Ad26.ZEBOV/MVA-BN-Filo to MENVEO^®^) has been followed by an age de-escalation component for paediatric participants.

The changes to the trial design affected many aspects of the study beyond what is typically encountered in the setup phase of a trial. As the trial design evolved, budgets underwent multiple revisions, and project plans changed significantly while the team waited for approvals, with associated amendments to orders and procurement of equipment and consumables. The expectations of stakeholders and community members had to be managed as the study enrolment target was significantly reduced which shrank the study’s geographical footprint from an initial district-wide catchment area, to just the inhabitants of two of the district’s main towns.

### Partnerships

The time pressures of operating during an outbreak influenced how partnerships in Sierra Leone were established, and the study was implemented by organisations which had not previously worked together. Partners in-country were needed quickly to provide local leadership and expertise, and to implement field activities for the trial. After discussions with Sierra Leone’s Ministry of Health and Sanitation and other stakeholders, a research partnership was established with the University of Sierra Leone’s College of Medicine and Allied Health Sciences. However, College of Medicine staff had limited experience of clinical trials and therefore significant technical support was required from the London School of Hygiene & Tropical Medicine and the trial sponsor.

Two non-governmental organisations also worked on the trial. Goal joined as the trial’s logistics partner, while World Vision were engaged to provide community engagement and IT support, amongst other activities. Clinical research is outside of the usual remit of these organisations, and therefore it was essential that all partners understood others’ requirements and limitations from the beginning of the project.

### Regulatory and ethics reviews

EBOVAC-Salone study approvals were required from the London School of Hygiene & Tropical Medicine Ethics Committee, the Sierra Leone Ethical and Scientific Review Committee, and the Pharmacy Board of Sierra Leone the national regulatory authority. During the Ebola epidemic, the African Vaccine Regulatory Forum, with support from the World Health Organization, established a platform for conducting joint reviews of clinical trial applications with input from international regulatory agencies. This aimed to achieve rapid comprehensive reviews of clinical trials in the context of potentially limited regulatory and ethics review capacity in the sub-Saharan African countries participating in Ebola vaccine trials.^12^ The EBOVAC-Salone study team engaged with the African Vaccine Regulatory Forum review process at a meeting in April 2015 that was also attended by the Pharmacy Board of Sierra Leone and the Sierra Leonean ethics committee.

During the epidemic, the ethics committees and the Pharmacy Board implemented an expedited review process for Ebola-related studies. Both Sierra Leonean authorities experienced a dramatic rise in workload in 2014–2015 as they were receiving frequent submissions for Ebola-related research. Despite this, the authorities continued to give thorough reviews and completed most reviews within 1 month following submission. The sponsor and study team engaged the authorities from the start of the project, to prepare them for the possibility of study design changes in response to the evolving epidemic.

### Ethical considerations

Shortly after the Ebola epidemic was declared a Public Health Emergency of International Concern, the World Health Organization established a panel of experts to discuss ethical questions relating to the use of unproven interventions, with discussions held around appropriate trial designs during an outbreak.^13^ Both the World Health Organization’s ethics panel and a group of expert anthropologists issued guidance to research groups planning to conduct clinical trials during the epidemic.^14^

An overarching ethical concern was that clinical research should not impact on the health system or the emergency response. The World Health Organization coordinated prioritisation of the most promising research, so as not to overburden the health systems in the affected countries.^15^ In Sierra Leone, planning for the EBOVAC-Salone study was undertaken in partnership with the College of Medicine and Allied Health Sciences, and a criterion was set that healthcare workers involved in the emergency response would not be recruited to the study team. Senior staff were therefore recruited from overseas to provide technical support, while also building the experience of more junior national staff who had not previously been involved in clinical research.

The question of whether trials should be placebo-controlled was particularly contentious,^16–19^ although not a new debate.^19^ During initial discussions held in Sierra Leone in early 2015 by the EBOVAC-Salone team, the country’s authorities did not favour a randomised placebo-controlled trial, and this ethical concern informed the study design as discussed above. In common with some other Ebola vaccine trials taking place in the region, the research team therefore developed an innovative trial design to study efficacy without the use of a placebo. This trial design was never implemented, because the epidemic’s decline made it impossible to establish vaccine efficacy before this study could be initiated.

Further ethical issues became apparent once the study began. EBOVAC-Salone social science research indicated that one of the motivating factors for participation in the trial was an expectation that the vaccine would be effective. The research team addressed this by placing additional emphasis on the investigational nature of the vaccine during community outreach activities and the informed consent process. The latter included a test which participants had to pass before being enrolled to demonstrate their understanding of the trial.

Initial participant recruitment took place prior to the end of the epidemic, and demand to participate in the study at that time was greater than the number of participants needed. To ensure fair subject selection, a lottery system was devised, whereby households were selected and prioritised in an open ballot.^20^ An age de-escalation approach was adopted with safety data being reviewed by an independent data monitoring committee prior to the enrolment of each cohort of children. Pregnant women were excluded because of concerns over the safety of viral vaccines given during pregnancy. This exclusion criterion was common to all Ebola vaccine trials in the region, although some authors have argued that, in future, pregnant women should not be denied the potential benefits of participating in clinical research in an outbreak of such high mortality.^21^

### Communications and engagement

The normal processes of engaging with communities prior to a trial of a new investigational medical product were given an additional layer of complexity by the Ebola outbreak. The challenges of community engagement and building trust and awareness in a setting with no experience of clinical trials, such as Kambia, were exacerbated by the urgency to commence the research.

Formative, qualitative research into community dynamics and perceptions around Ebola and the clinical trial were especially important during a period that was characterised by a lack of trust in both national and international organisations.^22^ This research, and ongoing tracking of rumours and concerns, helped target engagement approaches and community messaging. For example, a rumour that the vaccine trial was bringing Ebola back to the community, nicknamed ‘Ebola Phase Two’, was identified by the social scientists. The community liaison team responded by visiting the market where the rumour had originated and initiating meetings to address the concerns with the support of pre-briefed local stakeholders.

Establishing the study while the emergency response infrastructure was in place was challenging for the study’s community engagement in some ways, but facilitated it in others. The Ebola response centres brought stakeholders together, providing an efficient mechanism through which these individuals could be briefed. Because many stakeholders were already engaged in the fight against Ebola, they were often particularly receptive to research teams.

However, in an environment where people were desperate for solutions, the study’s community engagement had to clearly differentiate the research project from the Ebola response to avoid possible misinterpretations that the trial was delivering a licenced vaccine as part of the humanitarian response.

### Participant recruitment

During the outbreak, community interest in participating in the first stage of the study was high. EBOVAC’s social science research indicated a range of motivating factors behind participation in the early stages of the trial, including the notion of ‘sacrifice’ or duty as a citizen; and hope or belief in the power of the vaccine to prevent Ebola.

Contrary to the experience of some groups conducting research during the outbreak, generous financial compensation was not necessary to stimulate participation in the trial in Kambia.^8^ Study partners sought advice from the national ethics committee before agreeing to rates that would fairly recompense a participant’s time and transport without providing undue inducement, based on estimated lost earnings and transport costs to and from the clinic.

### Human resources

The limited pool of clinicians in Sierra Leone was severely affected by the outbreak as Ebola-related mortality among health workers was particularly high.^23^ Prior to the epidemic, Sierra Leone ranked fifth from bottom among 193 countries in terms of numbers of physicians per head of population.^24^ As discussed above, so as not to further weaken the wider health system, the research team committed not to recruit any healthcare workers employed in the government health system or involved in the Ebola response without the approval of the appropriate authorities.

Because limited clinical research had been conducted in Sierra Leone prior to the Ebola epidemic, there were very few individuals in the country with appropriate research experience, and those that had this were generally involved in other Ebola-related studies. Technical and scientific support was therefore provided to the in-country team, facilitated by the London School of Hygiene & Tropical Medicine’s response to the epidemic that allowed staff to temporarily leave their current post to work on the Ebola response.

It proved challenging to attract suitably qualified and experienced applicants to work in longer term positions on the trial. This may have been due to the perceived risk of working in an Ebola-affected country, because the trial site was in a relatively undeveloped district, and because people with the relevant skills were not available to apply for posts at short notice. In addition, the London School of Hygiene & Tropical Medicine is not an emergency response organisation and therefore did not provide hardship or risk allowances to staff employed on the study.

### Operational issues

Many operational challenges arose because the epidemic occurred in a region with a low level of development; Sierra Leone remains among the world’s poorest countries and suffers from poor infrastructure.^25^ Outside an outbreak situation, a rural district like Kambia would not be a natural choice for an urgent trial due to the extensive requirements and costs of delivering a Good Clinical Practice–compliant study in this setting.

Kambia is not on the national power grid and therefore a 24 hour generator-based power supply was installed for vaccine storage, offices, and laboratories, and a fuel supply chain and fuel store were established. While it was possible to rent, renovate, and equip existing buildings for the research clinics and offices, it was necessary to construct a vaccine storage facility and establish a research laboratory. Logistics became hugely time-consuming, necessitating dedicated operations staff to oversee construction, electrical installations, and laboratory setup. In common with many emergency situations, the cost of accommodation, rental and construction of buildings, and goods such as furniture rose substantially with the arrival of Ebola response partners.

Emergency public health restrictions implemented to curtail the spread of Ebola affected the establishment of the study. For example, nightly curfews limited working hours, including on the study-related construction and renovations, and restricted the movement of people and vehicles throughout the country.

However, engagement with the Ebola response authorities allowed the team to gain special dispensation to assist with setting up the research project. These included securing permits to allow team members to travel during curfew periods in Kambia; and a blanket exemption on import tax for equipment and consumables.

### Post-outbreak transition

The EBOVAC-Salone trial is now in the age de-escalation stage of the trial and has experienced the transition from working in an outbreak setting to a post-outbreak setting, and the return to ‘business as usual’.

The response infrastructure in Sierra Leone was quickly dismantled once the outbreak ended and the stakeholder landscape changed almost overnight as roles and responsibilities at national and district level reverted to their pre-Ebola position. The study team had to reassess the potential influence of various individuals or institutions on the trial and adjust relationships accordingly.

The dispensations related to the emergency situation were suspended rapidly once the outbreak was declared over. This included the waiver on importation taxes, which was not clearly communicated to the research team, thereby complicating the clearing of a subsequent shipment.

After the outbreak had ended, interest in study participation decreased, in part due to a shift in the risk/benefit perception of members of the community, many of whom felt that Ebola had been eradicated permanently. The household lottery method for recruiting participants was therefore expanded and a broader ‘first come, first served’ approach was adopted.

Post-outbreak, many of the logistical challenges remain such as the lack of a mains power supply, and staff recruitment and retention to a relatively remote area of the country. Despite these challenges, the study completed enrolment of adult participants in October 2016 and is recruiting participants into the age de-escalation component of the trial. Over 100 Sierra Leonean study personnel, including some Kambia Government Hospital staff, have been trained in essential research skills and clinical management. This will hopefully provide a legacy and a platform on which to build further research in the district.

## Recommendations

Following the experiences of establishing the EBOVAC-Salone trial and the lessons learned between 2014 and 2017, we have a number of recommendations for teams aiming to conduct research during epidemics.

### Trial design

Research teams, funders, regulatory authorities, and ethics committees should be flexible and prepared for additional workloads when initiating and implementing clinical trials in outbreak settings, as trial designs may need to be adapted and submitted for review several times over a short period. Modelling can be utilised to predict trends and help identify research sites as the epidemic unfolds. For trials of investigational medical products, study design changes may continue after an epidemic ends as the advice of international regulatory authorities may also change if the results of the trial are part of the submission for licensure. There is now a substantial body of information regarding the acceptability of outbreak-specific trial designs from the Ebola epidemic that could be used to inform template trial designs in advance of future outbreaks.

### Partnerships

Identifying partnerships with established, on-the-ground organisations may require urgent networking and meetings to build trust and agree goals if partners have not worked together before. Linkages with organisations that are not necessarily ‘classical’ research partners can be extremely valuable for implementing research in an emergency situation.

### Regulatory and ethics reviews

Ethical and regulatory authorities in countries with limited experience of clinical trials or other studies conducted during an epidemic may need to be supported by regional and international bodies to develop guidelines for study submissions during epidemics. Additional funding may be required to increase capacity for urgent reviews. The African Vaccine Regulatory Forum and similar bodies can usefully support ethics committees and regulators to facilitate the review and approval of clinical trials conducted during an epidemic if utilised at the right time, and sponsors and researchers should engage early with such a forum.

### Ethical considerations

Experiences around ethical issues pertaining to the design and implementation of clinical trials during the Ebola epidemic should be considered for future outbreaks. As soon as an outbreak is declared, the potential need for appropriate research should be considered as an integral part of the response planning to ensure that possible ethical concerns come to the fore and are debated at the first possible opportunity. Research must not have a detrimental impact on either the outbreak response or health systems.

### Communications and engagement

Social scientists should be considered an integral part of any large clinical trial team, particularly for trials in an outbreak situation where it is vital to understand community dynamics and concerns that may change frequently. During an outbreak, additional efforts may be needed to explain and reinforce the distinction between an investigational medical product and a licenced treatment or vaccine, especially when fear of infection may be more likely to influence participant decision-making.

### Participant recruitment

Interest in participation may fluctuate based on community perceptions of risk associated with the outbreak and the benefits of participation, and recruitment strategies may therefore need to be adapted as epidemics evolve or wane.

### Human resources

Researchers should engage local health authorities to establish guidelines for recruiting staff to prevent weakening outbreak responses or health systems. Recruitment of certain cadres of staff from countries or areas not affected by the outbreak may need to be considered, at least temporarily.

### Operational issues

During an outbreak, researchers may need to choose study sites that are not ideal in order to conduct the study in a suitable target population while avoiding other research projects. Researchers should respect emergency restrictions, but may be able to explore dispensations with the public health authorities if they do not pose a risk to outbreak control activities. Researchers should establish what happens to these dispensations should the outbreak end.

### Post-outbreak transition

Researchers should ensure that they identify and engage both routine and response structures and government regulations to help ensure continuity of support and trial activities once an outbreak is over.

## Conclusion

Many challenges have been identified while setting up and implementing clinical trials during the West African Ebola epidemic. We found that, despite implementing a study with new research partners in a district with poor infrastructure and health services, where clinical research is in its infancy, interest in participation was high. The EBOVAC-Salone study has enrolled a total of 443 adults, 192 adolescents, and 132 children between October 2015 and December 2017, and the study continues to enrol younger cohorts of participants. However, in common with other research groups, we recommend that research should be more closely involved in outbreak response planning, which could expedite the establishment of research projects and maximise their chances of success.

While many of the lessons learned from the EBOVAC-Salone experience will be specific to the context of the Ebola outbreak in Sierra Leone, it is hoped that the principles behind them are transferable to research teams in other outbreak situations. These recommendations could be added to programmes and strategies to improve the responses to future epidemics.
